# Method for real-time prediction of cutter wear during shield tunnelling: A new wear rate index and MCNN-GRU

**DOI:** 10.1016/j.mex.2023.102017

**Published:** 2023-01-18

**Authors:** Nan Zhang, Lin-Shuang Zhao

**Affiliations:** a Department of Civil Engineering, School of Naval Architecture, Ocean, and Civil Engineering, Shanghai Jiao Tong University, Shanghai 200240, China; b Department of Civil and Environmental Engineering, College of Engineering, Shantou University, Shantou 515063, China

**Keywords:** 1D-CNN, Wear rate index, Gated recurrent unit, Wear prediction, Integrated method based on a new index and CNN-GRU

## Abstract

Cutter wear is one of the key factors influencing construction efficiency during shield tunnelling. Prediction of cutter wear can improve construction efficiency by reducing the times of cutter inspections in engineering practice. Evaluation of cutter life is vital for cutter wear prediction, however, existing cutter life indices can only estimate the health condition of all cutters on cutterhead on a holistic basis. A new index was proposed to evaluate cutter wear located at a specific installation position on cutterhead. A deep learning model integrating the index was developed for the estimation of accumulated cutter wear during real time shield tunnelling. The new index can be obtained by monitored field parameters and can predict cutter wear with historical wear patterns. The input and output data samples were reshaped for multi-step prediction. A shield tunnelling section in Guangzhou weathered granite was used for validation. The proposed method can help reduce the cost of cutter replacement by reducing the times of machine interventions. The method article is a companion paper to the original article [1].

• Proposed index for prediction of cutter wear rate.
• Deep learning model of 1D-CNN and GRU.
• Multi-step cutter wear prediction.

## Graphical abstract

**Figure:**
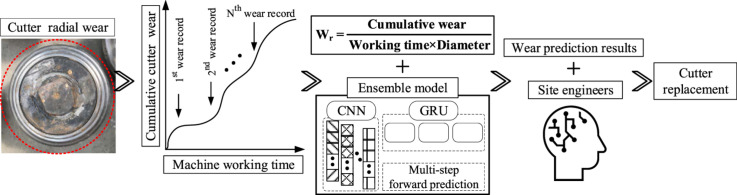

Specifications table

**Table:** 

Subject area:	Engineering
More specific subject area:	Shield tunnelling
Name of your method:	Integrated method based on a new index and CNN-GRU
Name and reference of original method:	Jarrett, K., Kavukcuoglu, K., Ranzato, M., Lecun, Y. (2009). What is the best multi-stage architecture for object recognition? in: IEEE 12th International Conference on Computer Vision, 2009, 2146–2153, https://doi.org/10.1109/ICCV.2009.5459469. [2] Cho, K., Van Merrienboer, B., Gulcehre, C., Bahdanau, D., Bougares, F., Schwenk, H., Bengio, Y. (2014). Learning phrase representations using RNN encoder-decoder for statistical machine translation. arXiv preprint, https://arxiv.org/abs/1406.1078. [3]
Resource availability:	DOI: https://doi.org/10.1016/j.tust.2022.104830.

**Method details**

## Index for evaluation of cutter wear

### The proposed index

Wear of cutters replaced from a certain position on cutterhead was defined in Eq. (1):

(1) $$W_{r}=w/\left(t\times d\right)$$

where *W_r_* is the proposed index for estimation of cutter wear rate, *w* is the cumulative wear of cutters in millimeter (mm), *t* is the working time between cutter and ground during shield tunnelling in minute (min), *d* is the cutter ring diameter in (mm).

Cutter wear records were derived from maintenance sheets during shield machine interventions where cutter radial wear was measured manually with customized calibrators. For a specific tunnelling case, cutter ring diameters are usually determined. Cutter ring diameters of 432 mm (17 inches) and 483 mm (19 inches) are used by the overwhelming majority of shield machines [4]. The accumulated cutter wear at a certain installation position equals to the addition of wear of each worn cutter replaced at that position. The raw data included non-working time data and data noise which needs to be eliminated. Non-working time data was filtered using Eq. (2) and (3) [5,6]:

(2) G=g(P)×g(A)×g(C)

(3) $$g\left(x\right)=\left\{ \begin{array}{c} 1,\;\\ 0, \end{array}\right.\begin{array}{c} x\neq 0\\ x=0 \end{array}$$

Where x is the collected boring data samples, *P* is penetration depth per revolution, *A* is the advancing speed of the shield machine, and *C* is cutterhead rotation speed. If G equals to zero, the corresponding data sample will be removed. Otherwise, the data was regarded as working time data which will be reserved for follow-up calculations. The data noise in the working time data was then filtered with the assumption that the boring data of each ring followed the Gaussian distribution. Data within 68% confidence interval was qualified for calculation of machine working time. Data noise can be removed by Eq. (4) [7]:

(4) $$g_{j}\in \left(\mu_{j}^{i}-\sigma_{j}^{i},\mu_{j}^{i}+\sigma_{j}^{i}\right),\;1\leq i\leq m;\;\forall \;j\in \left\{P,\;A,C,T_{f},\;T_{o}\right\}$$

Where *i* and *j* are boring parameters and tunnel rings, respectively, μ is the mean value, σ is the standard deviation, $g_{j}$ is data samples, *m* is the number of rings, $T_{f}$ and $T_{o}$ are thrust and torque, respectively.

Working time was related to the number of remaining data in that ring after the filter of empty data and data noise elimination. Working time in Eq. (1) can be determined by recording the frequency of boring parameters which can be adjusted manually during tunnelling [8]. For example, if boring data was recorded at a one-minute interval, working time equals to the number of remaining data. If boring data was recorded every 30 seconds, working time equals to half of the number of remaining data.

The wear data was recorded during cutter inspections which were conducted at regular intervals. Linear interpolation, which was used to calculate the accumulated cutter wear of a certain cutter between two adjacent wear records, is formulated in Eq. (5):

(5) $$W_{a}=\frac{T}{T_{n}}\times \;\left(W_{n}-W_{n-1}\right)+W_{n-1}$$

Where, *W_a_* is the accumulated cutter wear of current time, *T* is the working time of the shield machine from the last cutter wear record, *T*_n_ equals to the construction time between the last wear record and current wear record, *n* equals to the number of wear records, *W*_n_ and *W*_n-1_ are the accumulated wear until current wear record and last wear record, respectively.

## Deep learning model

### Data preparation

The input parameters included boring parameters, covered depth, and the proposed index. Detailed parameter settings and statistical distributions of the boring parameters can be found in the companion paper [1]. The boring data samples constituted a data matrix which is presented in Eq. (6):

(6) $$P={\left[P_{i\times j}\right]}_{n\times m}=\left[\begin{array}{cc} \begin{array}{ccc} p_{1}^{1} & p_{1}^{2} & \cdots \\ p_{2}^{1} & p_{2}^{2} & \cdots \\ \vdots & \vdots & \ddots \end{array} & \begin{array}{ccc} p_{1}^{i} & \cdots & p_{1}^{m}\\ p_{2}^{i} & \cdots & p_{2}^{m}\\ \cdots & \cdots & \vdots \end{array}\\ \begin{array}{ccc} p_{j}^{1} & p_{j}^{2} & \vdots \\ \vdots & \vdots & \vdots \\ p_{n}^{1} & p_{n}^{2} & \cdots \end{array} & \begin{array}{ccc} p_{j}^{i} & \cdots & p_{j}^{m}\\ \vdots & \ddots & \vdots \\ p_{n}^{i} & \cdots & p_{n}^{m} \end{array} \end{array}\right]$$

Where *m* is the number of boring parameters, and *n* is the number of data samples. $p_{j}^{i}$ is the *i*th boring parameter of data sample *j*. Each row in Eq. (6) is a data sample recorded at a one-minute interval. As cutter inspections can be conducted at any time during tunnelling of one ring, cutter wear should be recorded to obtain the exact time, other than the ring number, when the cutter was replaced. Then, a logarithmic form of the proposed index was fitted into the data matrix according to time stamps. The complete data matrix for the prediction of a certain cutter is shown in Eq. (7):

(7) $$P^{\prime }={\left[P_{i\times j},W_{r}\right]}_{n\times \;\left(m+1\right)}=\left[\begin{array}{cc} \begin{array}{cc} \begin{array}{ccc} p_{1}^{1} & p_{1}^{2} & \cdots \\ p_{2}^{1} & p_{2}^{2} & \cdots \\ \vdots & \vdots & \ddots \end{array} & \begin{array}{ccc} p_{1}^{i} & \cdots & p_{1}^{m}\\ p_{2}^{i} & \cdots & p_{2}^{m}\\ \cdots & \cdots & \vdots \end{array}\\ \begin{array}{ccc} p_{j}^{1} & p_{j}^{2} & \vdots \\ \vdots & \vdots & \vdots \\ p_{n}^{1} & p_{n}^{2} & \cdots \end{array} & \begin{array}{ccc} p_{j}^{i} & \cdots & p_{j}^{m}\\ \vdots & \ddots & \vdots \\ p_{n}^{i} & \cdots & p_{n}^{m} \end{array} \end{array} & \begin{array}{c} \begin{array}{c} \log W_{r1}\\ \log W_{r2}\\ \vdots \end{array}\\ \begin{array}{c} \log W_{rj}\\ \vdots \\ \log W_{rn} \end{array} \end{array} \end{array}\right]$$

Where $\log W_{rj}$ is wear rate index based on Eq. (1) and Eq. (5). Following the time series in Eq. (7), around 80% of data samples were used as training dataset, while the remaining 20% was used for validation. Since the number of cutter replacements of cutters with different installation radii was different, the ratio of training data to test data was determined to be around 4:1. The data samples in Eq. (7) were then limited within [0, 1] by min-max normalization to accelerate model convergence.

### Reshape of input and output data

After data preparation, the input was shaped into a tensor with the shape of the data sample length, time step, and input dimension. The time step determined the number of time intervals used in one data sample. The output was programmed into [sample size, leads]. The leads are the number of time intervals predicted ahead of the current time. After reshaping of input and output data samples, the length of the input array can be calculated by Eq. (8):

(8) L=N×T×D

Where *L* is the length of the input array, *N* represents the number of input parameters, *T* represents the time step, and *D* represents the input dimensions.

### Integration of proposed *W_r_* and deep learning model

The integrated deep learning model consists of a convolution layer, a max pooling layer, a gated recurrent unit (GRU) layer, and a fully connected layer [9], [10], [11]. The proposed index, *W_r_*, was used as the output parameter of the deep learning model. The mean squared error between predicted and measured *W_r_* was used as the loss function of the model which was presented in Eq. (9).

(9) $$Loss=\frac{1}{n}\sum_{j=1}^{n}{\left(\hat{W_{rj}}-W_{rj}\right)}^{2}$$

**Fig. 1:**
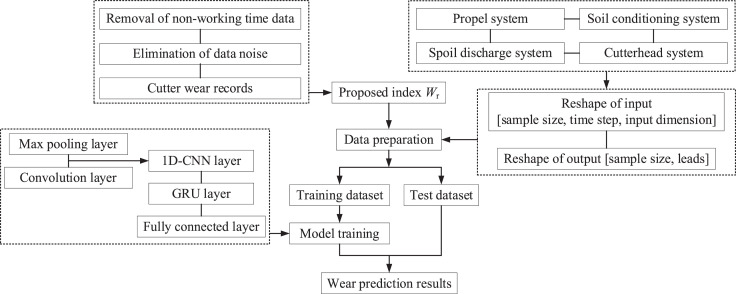
Flowchart of real-time prediction of cutter wear.

Where *n* represents the number of data samples in one batch size, $\hat{W_{rj}}$ is the predicted wear rate index, $W_{rj}$ is the measured wear rate index. The parameters of the deep learning model to be optimized were trained jointly by the Adam algorithm [12,13] during the model training process. Fig. 1 shows the flowchart of the proposed method.

## Computational tool

Keras, programmed with Python, was used for model establishment [14,15]. TensorFlow was used as the backend. Table A1 shows the pseudocode for multi-step prediction. The code includes reshaping input and output data samples for multi-step cutter wear prediction and interpretation of prediction results. Table A2 shows the pseudocode for the construction of the CNN-GRU model based on Keras. The code comprises of development of model architecture and the determination of model hyperparameters. The model was trained and tested using an Intel Xeon E5-2650 v2 @ 2.6 GHz CPU. The following steps were used for the prediction of cutter wear during shield tunnelling in real time.

(1) Empty data removal and elimination of data noise. Users should determine the cutter number for wear prediction. Then, training and test datasets which contain boring parameters obtained from the auto-acquisition system of the shield machine should be prepared. Non-working time data can be deleted based on Eq. (2) and Eq. (3). Then, data noise should be filtered using Eq. (4). It should be noted that Eq. (4) is case specific and can be influenced by the characteristics of the distribution of parameters recorded by the shield machine during tunnelling of one ring. Users should eliminate the influence of data which represents the non-working time between cutter and ground.
(2) Accumulated wear of the selected cutter should be calculated at each cutter inspection position [16]. Then, accumulated wear data can be integrated with boring data according to time stamps. Cutter wear conditions between adjacent cutter inspections can be obtained by Eq. (5). The wear rate index can be determined by Eq. (1).
(3) Preparation of training and test data. The input of the hybrid model included cutterhead parameters, soil conditioning parameters, power parameters, and spoil discharge parameters. Specifically, covered depth and tunnel face pressure was used to present variations of ground formations [17,18]. The result of the wear rate index should be transformed into a logarithmic form. One wear record, which is the critical timeline for grouping training and test datasets with a ratio of around 4:1, should be determined. Data samples before the selected wear record are used for model training while the rest is for model validation. The number of data samples in one input array can be calculated with Eq. (8) while the length of output equals to the leads. Time steps and leads can be adjusted by users according to the time needed for the construction of one ring in different cases for real time prediction.
(4) Model construction. The reshaped input data samples were first inputted into a convolutional layer where the convolution kernel scrolled on the input data samples with a determined stride. The output of the convolutional layer was down-sampled in the max pooling layer. The GRU layer was then used to consider the historical wear information of the selected cutter and to learn new wear characteristics when encountering new types of ground. The results of the size of the input array and output array determined the number of neurons in the input and output layers, respectively. Other model architectures and hyperparameters can be determined by trial and error.
(5) Model training. Eq. (9) was used as a loss function of the deep learning model during model training. Then, the model can yield prediction results of the selected cutter ahead of the current time. To predict cutter wear with multi-step results, a multi-step prediction strategy was developed. Five data samples, i.e., five rows in Eq. (7), were used as input. Three proposed *W_r_* after the input were used as output. The next input consisted of the last two data samples from the previous input and three new data samples after the previous input. The next three proposed *W_r_* were the next output. The training data was used to calibrate the intelligent model and the computational cost was 285s.
(6) The model with optimized trainable parameters was then used for the prediction of cutter wear conditions. Accumulated cutter wear of the selected cutter can be obtained using Eq. (1) with known working time between cutter and strata. Wear can be calculated by subtracting accumulated wear until the last wear records from the predicted result.

## Method validation

**Fig. 2:**
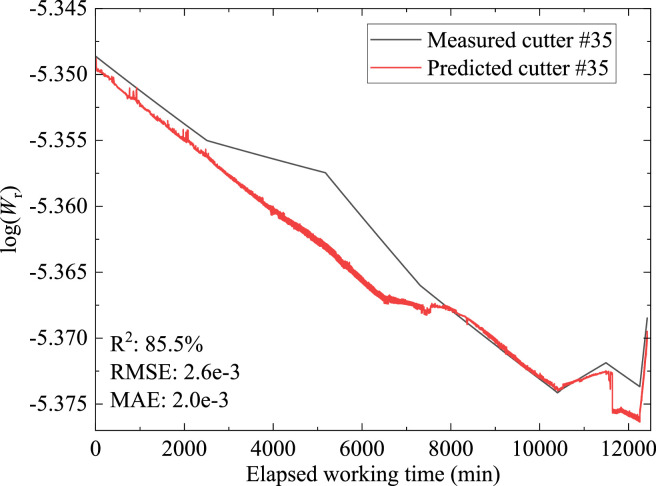
Comparison between predicted and measured log (*W*_r_) of cutter number 35.

**Fig. 3:**
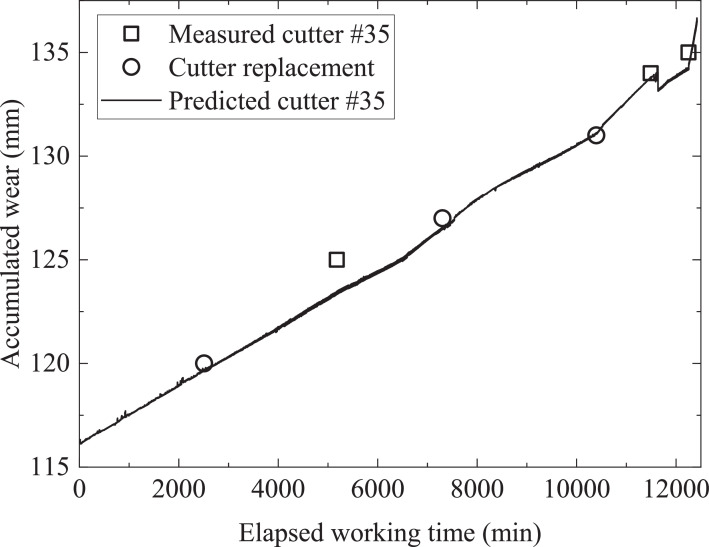
Prediction result of accumulated wear of cutter number 35.

Most existing indices cope with the health conditions of all cutters on cutterhead and reflect cutter life in terms of cutter consumptions or cutter radial wear after excavation of a certain distance [19]. The proposed method introduced a new index that can be integrated with deep neural networks for the prediction of cutter wear in real time. The new wear rate index integrated with the hybrid deep neural network was validated by field data from an earth pressure balance tunnelling case in Guangzhou city, China. Single-ring disc cutter with a diameter of 483 mm was used for excavation. Cutterhead specifications and cutter distribution can be found in the companion paper [1]. Fig. 2 shows the predicted and measured Log (*W*_r_) of cutter number 35. Wear conditions of cutter number 35 can be calculated using Eq. (1). Fig. 3 shows the wear increase of cutter number 35. The results showed the method can predict variations of cutter wear during real time excavation successfully. The coefficient of determination (R^2^), root mean squared error (RMSE), and mean absolute error (MAE) are 85.5%, 2.6e-3, and 2e-3, respectively. Comparison with other deep neural networks including MCNN-LSTM, MCNN-RNN, and MCNN with similar model structure and prediction strategy proved the superiority of the proposed model [1]. The proposed index shows the impact of ground conditions, boring parameters, and cutter ring material and geometry on wear features as the shield machine advances. Wear characteristics obtained from previous tunnelling sections can be experience for follow-up excavation. The proposed method in this article is case specific due to different cutterhead layouts and cutter geometry in different shield tunnelling projects. Supplementary field data from different shield tunnelling cases should be used to calibrate the new index and deep learning model before implementation. For the future application of the method, a database that includes operational parameters and corresponding wear data derived from different geological conditions and shield machine specifications should be established. The determination of hyperparameters of deep learning models can be optimized with machine learning techniques which can help accelerate model convergence and increase model performance.

## CRediT authorship contribution statement

**Nan Zhang:** Data curation, Methodology, Investigation, Software, Writing – original draft. **Lin-Shuang Zhao:** Visualization, Writing – review & editing.

## Declaration of Competing Interest

The authors declare that they have no known competing financial interests or personal relationships that could have appeared to influence the work reported in this paper.

## Data Availability

Data will be made available on request.

## Appendix Supplementary materials

Image, application 1
