# Supplementary material for: Method for real-time prediction of cutter wear during shield tunnelling: A new wear rate index and MCNN-GRU
Source: MethodsX. 2023 Jan 18;10:102017. doi: 10.1016/j.mex.2023.102017 (PMC9880237; doi:10.1016/j.mex.2023.102017)
Supplement: Supplementary file 1 [file mmc1.docx]

**Supplementary material *and/or* additional information [OPTIONAL]**

Table A1. Pseudocode of multi-step prediction of cutter wear.

| **Algorithm** Reshape of input and output data samples and result extraction algorithm |
| --- |
| # Reshape of input data samples  **def** Reshape of input data samples:  **do** calculation of sample size and number of input parameters  **do** definition of input dimension  **do** reshsape input data samples  X = empty set  for i = 1, 4, 7, …, number of data samples **do**  tempa = reshaped data samples [i, i+1, …, (i+5)]  a = []  for j = 1, 2, …, size(tempa) **do**  for k = 1, 2, …, size (lines in tempa) **do**  fill a with (tempa [j, k])  fill X with a  return X  # Reshape of output data samples  **def** reshape of output data samples  Y = empty set  for i = 5, 8, 11, …, number of data samples **do**  tempb = reshaped data samples [i:(i+3), last line of reshaped data samples]  b = empty set  for j = 1, 2, …, length of tempb **do**  for k = 1, 2, …, dimensions of output **do**  fill b with (tempb [j, k])  fill Y with b  return Y  #Results extraction  **def** result extraction  invy = empty set  for i = 1, 2, …, rows of result **do**  for j = 1, 2, …, lines of result **do**  fill invy with (result [i, j])  return invy |

Table A2. Pseudocode of construction of CNN-GRU model based on Keras.

| **Algorithm** model construction algorithm |
| --- |
| **def** model construction:  **do** calculation of time steps of training set  **do** calculation of number of parameters of training set  **do** add 1D-CNN layer  **do** add GRU layer  **do** add fully connected layer  **do** add output layer  **do** determine optimizer and loss function  **do** determine epochs and batch size, and validation with test dataset  return model |
